# Copper-Copper Oxide Heterostructural Nanocrystals Anchored on g-C_3_N_4_ Nanosheets for Efficient Visible-Light-Driven Photo-Fenton-like Catalysis

**DOI:** 10.3390/molecules30010144

**Published:** 2025-01-02

**Authors:** Guangying Zhou, Fan Yang, Ximiao Zhu, Weihua Feng, Dongdong Chen, Jianzhang Fang

**Affiliations:** 1 School of Environment, South China Normal University, Guangzhou 510006, Chinazhuximiao@m.scnu.edu.cn (X.Z.); zhlnano@hotmail.com (W.F.); esydchen@mail.scut.edu.cn (D.C.); 2 Guangdong Provincial Key Laboratory of Chemical Pollution and Environmental Safety & MOE Key Laboratory of Theoretical Chemistry of Environment, South China Normal University, Guangzhou 510006, China

**Keywords:** Cu-Cu_2_O/g-C_3_N_4_, photo-Fenton reaction, surface plasmonic effect, coupled photocatalysis

## Abstract

The development of efficient and sustainable photocatalysts for wastewater treatment remains a critical challenge in environmental remediation. In this study, a ternary photocatalyst, Cu-Cu_2_O/g-C_3_N_4_, was synthesized by embedding copper-copper oxide heterostructural nanocrystals onto g-C_3_N_4_ nanosheets via a simple deposition method. Structural and optical characterization confirmed the successful formation of the heterostructure, which combines the narrow bandgap of Cu_2_O, the high stability of g-C_3_N_4_, and the surface plasmon resonance (SPR) effect of Cu nanoparticles. The photocatalytic performance was evaluated through the degradation of Rhodamine B (RhB) in a photo-Fenton-like reaction system under visible light irradiation. Among the catalysts tested, the 30 wt% Cu-Cu_2_O/g-C_3_N_4_ composite exhibited the highest catalytic efficiency, achieving a reaction rate constant approximately 3 times and 1.5 times higher than those of Cu-Cu_2_O and g-C_3_N_4_, respectively. Mechanistic studies suggest that the heterostructure facilitates efficient charge separation and promotes the reduction of Cu^2+^ to Cu^+^, thereby enhancing ∙OH radical generation. The catalyst also demonstrated excellent stability and reusability across a wide pH range. These findings provide a new strategy for designing highly efficient photocatalysts for organic pollutant degradation, contributing to the advancement of advanced oxidation processes for environmental applications.

## 1. Introduction

Over the past decades, advanced oxidation processes (AOPs) have attracted considerable attention in the removal of environmental organic pollutants in wastewater [1,2,3]. Among these, Fenton technology has emerged as one of the most widely utilized AOPs, owing to its efficiency in generating highly reactive hydroxyl radicals (∙OH) through the activation of hydrogen peroxide (H_2_O_2_) by Fe^2+^ ions [4]. However, the practical application of the traditional photo-Fenton reaction over Fe-based systems was limited by some factors, such as the narrow working pH range and the production of Fe-containing precipitates [5,6]. It is reported that the copper-based Fenton-like system shows strikingly similar redox properties as the iron-based Fenton system, and it can be used in a wide pH range of 3–9 [7,8]. Therefore, to solve problems of Fe-containing systems, a series of heterogeneous photo-Fenton catalysts based on inexpensive Cu metals have been developed in recent years; among them, the most representative one is a cuprous oxide (Cu_2_O) [9,10,11,12]. Cu_2_O possesses a narrow band gap of ca.2.0 eV, which can efficiently adsorb the solar visible light [13]; thus, it can be an excellent photocatalyst [14]. However, in the photo-Fenton reaction, Cu^+^ (i.e., catalytically active species) will be oxidized to Cu^2+^ by H_2_O_2_ and thus lose the catalytic activity [15]. That is to say, promoting the efficient reduction of Cu^2+^ to Cu^+^ is critical for enhancing the catalytic performance of Cu_2_O-based photo-Fenton systems.

Constructing composite photocatalyst systems has recently emerged as a compelling strategy to overcome these challenges. Specifically, in the composite configuration, photo-generated electrons can transform from photocatalysts to metal ions (i.e., Cu^2+^) with higher valance states and can thus promote the reduction process of Cu^2+^ to lower valance states (i.e., Cu^+^) [9]. The newly formed Cu^+^ will participate in the photo-Fenton reaction and result in the generation of much more reactive •OH species. Graphitic carbon nitride (g-C_3_N_4_) was considered to be one of the most important candidates for constructing heterojunctions due to its suitable band gap (i.e., 2.7 eV) and good chemical as well as thermal stability [16,17]. In addition, g-C_3_N_4_ is composed of two earth-abundant elements (i.e., C and N) and can be easily obtained via pyrolysis of urea or melamine [18]. In recent years, a series of g-C_3_N_4_-based systems were developed, for example, Ag_2_O/g-C_3_N_4_ [19], g-C_3_N_4_/CeO_2_ [20], and g-C_3_N_4_/bismuth-based materials [21,22]; these composites all showed better catalytic activity than the individual components. Therefore, the construction of Cu_2_O/g-C_3_N_4_ systems may be beneficial for the charge separation process and thus can result in the generation of many more photo-generated electrons.

Moreover, the noble metal (i.e., Au or Ag) could strongly absorb visible light due to the strong apparent local surface plasmon resonance (LSPR) effect, which can greatly enhance the optical absorption efficiency of photocatalysts [23]. According to the previous publications, both Ag-g-C_3_N_4_ [24] and Au-g-C_3_N_4_ [25] promoted good catalytic performance compared with the bulk g-C_3_N_4_ owing to the SPR effect. In addition, some reports reported that inexpensive metals (i.e., Cu or Bi) also display LSPR absorption in the visible light region [26,27,28]. In addition, in the composite systems, Cu or Bi NPs (i.e., Cu^0^ or Bi^0^) possess excellent conductive ability and low work functions; thus, they can promote the electron transfer process in the photocatalytic reaction [28,29]. Chen et al. demonstrated that the presence of Bi^0^ was beneficial for the degradation of Rhodamine B (RhB) over Bi_2_O_3_/g-C_3_N_4_ photocatalysts [28]. Cheng found that the photocatalytic activity of the Cu_2_O-based system was significantly improved due to the existence of Cu NPs [29].

In this study, the ternary Cu-Cu_2_O-g-C_3_N_4_ composite was prepared and characterized by a series of analytical techniques. The catalytic performance of the as-obtained catalysts for organic pollutants degradation in the H_2_O_2_-containing system under visible light irradiation was also investigated. In addition, the influences of initial solution pH and concentration of H_2_O_2_ on the performance of the ternary catalyst were evaluated. Finally, the catalytic mechanism of the photo-Fenton-like system (i.e., Cu-Cu_2_O-g-C_3_N_4_) was proposed to explain its exceptional enhancement in the degradation process of RhB.

## 2. Results

### 2.1. Characterizations

**Figure 1:**
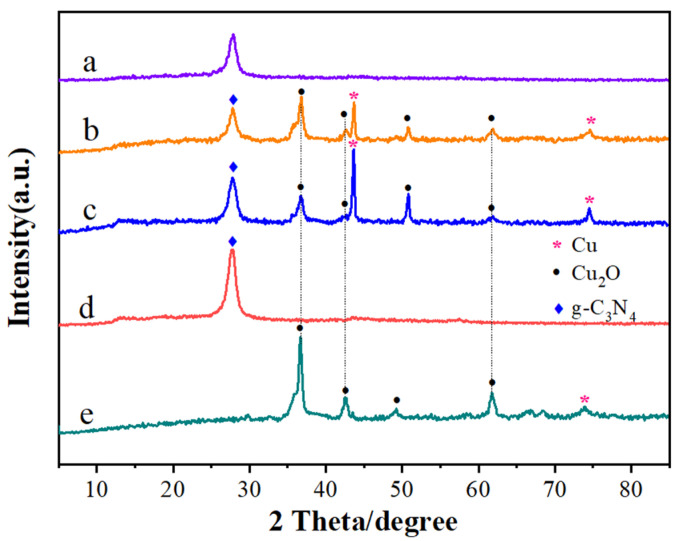
XRD patterns of the sample: (**a**) g-C_3_N_4_ nanosheets, (**b**) 50% Cu-Cu_2_O/g-C_3_N_4_, (**c**) 30% Cu-Cu_2_O/g-C_3_N_4_, (**d**) 5% Cu-Cu_2_O/g-C_3_N_4_, (**e**) Cu-Cu_2_O.

The diffraction patterns of the as-prepared Cu-Cu_2_O, g-C_3_N_4_, and Cu-Cu_2_O/g-C_3_N_4_ composites are shown in Figure 1. Apparently, the diffraction peaks that appeared at 27.8° could be in accordance with (002) planes of g-C_3_N_4_ [30]. The results indicated that the crystalline structure of g-C_3_N_4_ in the ternary Cu-Cu_2_O/g-C_3_N_4_ samples is complete. It can be observed that the characteristic peaks of Cu and Cu_2_O gradually appeared with the increase in Cu-Cu_2_O content. Specifically, the diffraction peaks located at 43.6°, 50.8°, and 74.4° match with Cu (JCDF-04-0836) and the other peaks located at 36.8° and 61.8°, which correspond to Cu_2_O (JCDF-050667) [29]. The results of the XRD patterns of (b) and (c) in Figure 1 suggested that the ternary Cu-Cu_2_O/g-C_3_N_4_ was successfully synthesized. Notably, the intensity of Cu in 30% Cu-Cu_2_O/g-C_3_N_4_ is higher than that of Cu in 50% Cu-Cu_2_O/g-C_3_N_4_. This is because the high-temperature calcination of hydrazine reduced metallic Cu at 200 °C (see experimental), which could result in a mixture of Cu and Cu_2_O, and the crystal of Cu_2_O is dominant in the binary phase, while the heterojunction interface between g-C_3_N_4_ and as-formed Cu can protect part of Cu crystals from oxidation in the calcination process (those Cu crystals that are anchored closely between the g-C_3_N_4_ sheets and oxidized adjacent Cu_2_O crystals).

**Figure 2:**
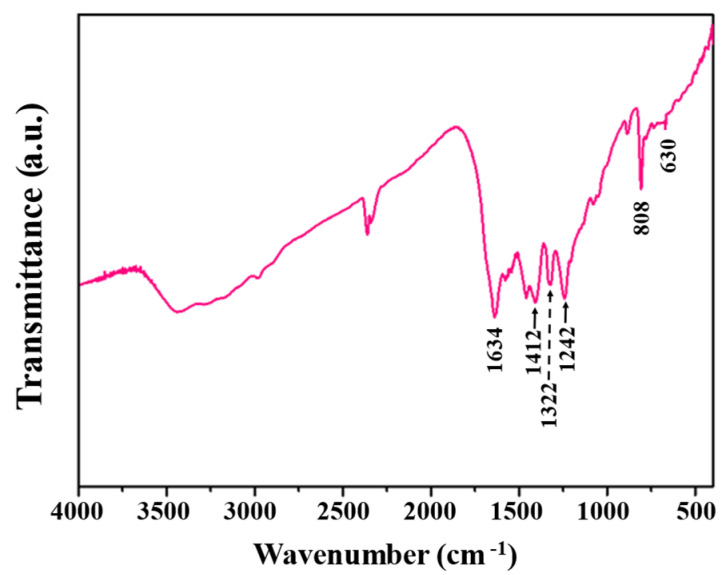
FTIR spectra of 30% Cu-Cu_2_O/g-C_3_N_4_.

The chemical composition of 30% Cu-Cu_2_O/g-C_3_N_4_ was further investigated by FT-IR measurements. The as-obtained spectrum is shown in Figure 2; it can be observed that there is a wide peak at 3000~3500 cm^−1^, which corresponds to the stretching vibration of N-H and the surface-absorbed water. In addition, the characteristic peaks at 1242, 1322, 1412, and 1634 cm^−1^ are ascribed to the stretching vibration of CN heterocycles. Moreover, the band at 808 cm^−1^ is attributed to the breaching mode of the s-triazine ring system [31]. As compared to pure g-C_3_N_4_, a new peak at 630 cm^−1^, which corresponds to the Cu (I)–O bond stretching vibration of Cu_2_O, has emerged [32]. These results indicated the presence of Cu_2_O and g-C_3_N_4_ in the Cu-Cu_2_O/g-C_3_N_4_ sample. However, no peak matching with the Cu nanoparticles could be found in the spectrum; this may be a result of the non-active properties of Cu^0^ in the infrared spectrum.

**Figure 3:**
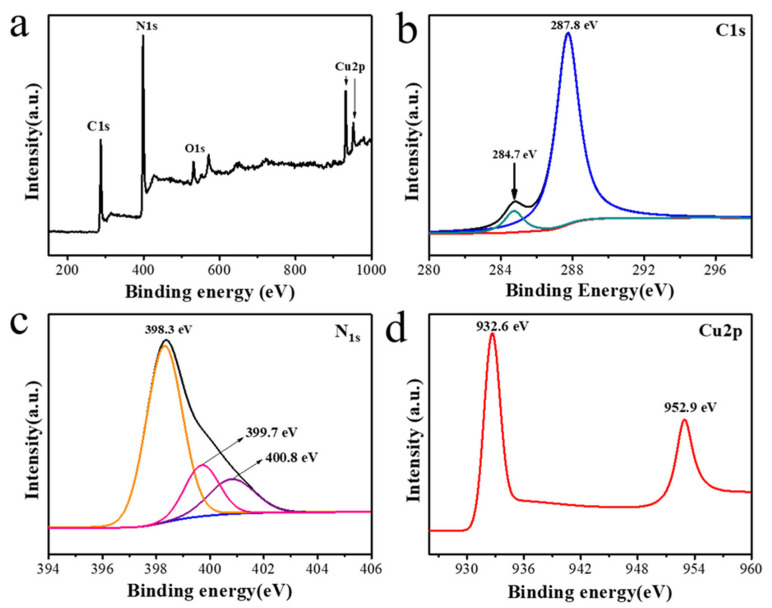
XPS spectra of 30% Cu-Cu_2_O/g-C_3_N_4_. (**a**) Survey spectrum, (**b**) C 1s, (**c**) N 1s, (**d**) Cu 2p.

To further understand the element composition and chemical states in the Cu-Cu_2_O/g-C_3_N_4_, the X-ray photoelectron spectroscopy (XPS) tests of the 30% Cu-Cu_2_O/g-C_3_N_4_ sample were conducted. As shown in Figure 3a, the XPS survey spectrum indicated that the ternary catalyst was composed of C, N, O, and Cu elements. Specifically, the high-resolution C 1s spectrum is shown in Figure 3b. The peak at 284.6 eV was corresponding to the C-C bond, and another major peak located at 287.8 eV was attributed to sp^3^-bonded C in C−N [33]. Deconvolution of the N 1s spectra allowed us to distinguish three N-containing compounds in the Cu-Cu_2_O/g-C_3_N_4_ sample (Figure 3c). Typically, the main peak that appeared at 398.3 eV was ascribed to sp^2^-N hybridized N-C=N, while the other two peaks at 399.7 eV and 400.8 eV could be attributed to amine groups (C-N-H) and tertiary nitrogen group (N-(C)_3_), respectively [34]. The combination of the results in Figure 3b,c further confirmed the presence of g-C_3_N_4_ in the ternary samples. The high-resolution of Cu 2p in Figure 3d was applied to test the Cu state. The characteristic peaks at 932.6 eV and 952.9 eV are attributed to Cu 2p^2/3^ and Cu 2p^1/2^, which are ascribed to Cu_2_O and Cu, respectively [34,35]. The result of the XPS analysis confirmed the co-existence of Cu, Cu_2_O, and g-C_3_N_4_ in the 30% Cu-Cu_2_O/g-C_3_N_4_ sample, which is in accordance with that of the XRD and FT-IR test.

**Figure 4:**
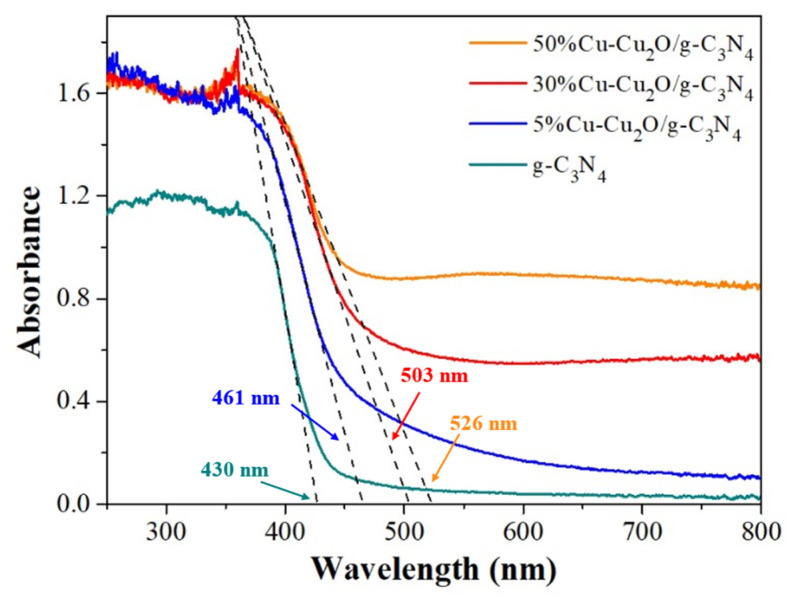
UV–Vis diffuse reflectance spectra of the as-prepared sample.

UV–Vis diffuse reflection spectra (DRS) were used to illustrate the optical properties of the as-prepared samples (Figure 4). According to previous publications, g-C_3_N_4_ possessed an absorption edge of 460 nm, matching the band gap of 2.70 eV [28]. As can be seen in Figure 4, the absorption edges of g-C_3_N_4_, 5%, 30%, and 50% Cu-Cu_2_O/g-C_3_N_4_ are 430, 461, 503, and 526 nm, respectively; thus, the bandgap energies can be calculated to be 2.88, 2.69, 2.47, and 2.36 eV [28]. Compared with the pure g-C_3_N_4_, the absorption spectra of the composite samples exhibited an obvious red shift, indicating that the absorption of the heterostructures in the visible light range was enhanced. In addition, with the increased content of Cu-Cu_2_O, the intensity of the absorption peak for the sample was improved. The enhanced photo-response ability might be attributed to the localized surface plasmon resonance (LSPR) of the Cu nanoparticles and the strong light absorption nature of Cu_2_O. Overall, the Cu-Cu_2_O/g-C_3_N_4_ composites exhibited stronger visible light absorption performances than the pristine g-C_3_N_4_ and gradually increased with the content of Cu-Cu_2_O.

**Figure 5:**
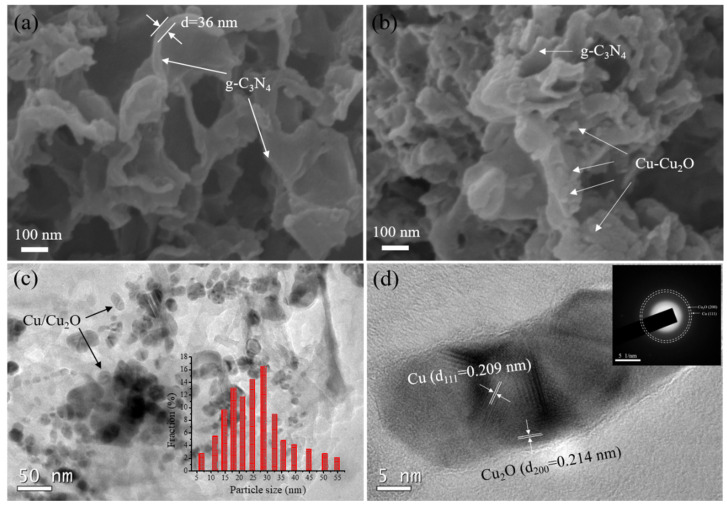
SEM images of pure g-C_3_N_4_ (**a**) and 30% Cu-Cu_2_O/g-C_3_N_4_ (**b**), TEM images of 30% Cu-Cu_2_O/g-C_3_N_4_ with an inset showing particle size distribution for Cu-Cu_2_O (**c**), and HR-TEM image of a single particle of Cu-Cu_2_O (**d**) in 30% Cu-Cu_2_O/g-C_3_N_4_ composite, the inset is the corresponding SAED pattern.

The morphology and microstructure of the as-prepared samples are characterized by SEM and TEM measurements (Figure 5). Pure g-C_3_N_4_, showing a lamellar structure with a thickness of the sheet of ~36 nm, is clearly observed (Figure 5a); after deposition of the copper oxide nanoparticles, the surface of g-C_3_N_4_ becomes rough, and discrete “dots” on the surface can be seen (Figure 5b). The TEM technique gives more evidence on the dispersion of the copper oxide on g-C_3_N_4_ nanosheets, and the size distribution of this copper oxide is mainly in the range of 15–35 nm (inset in Figure 5c). Further HR-TEM analysis was performed to make the nature of these anchored nanoparticles clear, as shown in Figure 5d. Two different lattice spacings of 0.2088 nm and 0.2135 nm could be determined from the single particle (Figure 5d); the former one is ascribed to the (111) crystallographic plane of Cu metal and the latter one corresponds to the (200) crystallographic of the Cu_2_O species. In addition, the SAED pattern in the inset of Figure 5d also confirmed the crystal information of the as-prepared ternary heterostructures. Besides this, a close interface between g-C_3_N_4_ and Cu-Cu_2_O could be observed in the Cu-Cu_2_O/g-C_3_N_4_ nanostructures. It is well-accepted that tight contact is beneficial for charge transfer and promotes the photogenerated charge separation in the Cu-Cu_2_O/g-C_3_N_4_ heterostructures [36].

### 2.2. Performance Evaluation

**Figure 6:**
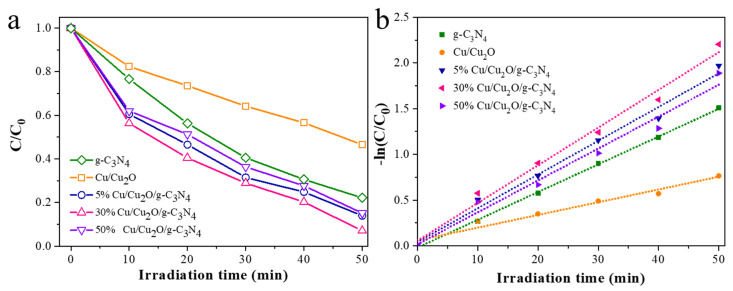
RhB degradation over the different catalysts in the presence of H_2_O_2_ under visible light irradiation for pure g-C_3_N_4_ nanosheets, Cu-Cu_2_O, without catalyst, 5% Cu-Cu_2_O/g-C_3_N_4_, 30% Cu-Cu_2_O/g-C_3_N_4_, and 50% Cu-Cu_2_O/g-C_3_N_4_ (**a**), and their kinetic plots (**b**).

RhB was chosen as a model pollutant to test the photo-Fenton catalytic performance of the catalysts. The RhB degradation curves and their kinetic plots of the-synthesized samples are shown in Figure 6. After exploratory experiments, it was found that the addition of H_2_O_2_ alone, visible light alone, or visible light when H_2_O_2_ was added had no degradation effect on RhB. In the photo-Fenton reactions, the initial pH of the RhB solution was set as 5.6. In the presence of H_2_O_2_, the Cu-Cu_2_O and g-C_3_N_4_ showed poor photocatalytic activity, achieving degradation rates of 46% and 78% after 50 min under visible light irradiation, respectively (Figure 6a).

Unlike the separated components, copper-copper oxide heterostructural nanocrystals embedded on g-C_3_N_4_ can promote photocatalytic activity. Furthermore, among other Cu-Cu_2_O/g-C_3_N_4_ samples, the ternary one with 30% wt. Cu-Cu_2_O showed the best catalytic degradation efficiency, achieving a degradation rate of about 90%. However, when the content of Cu-Cu_2_O further increased, the photo-Fenton performance of the catalyst decreased. To more directly and accurately understand the photo-Fenton catalytic reaction processes, the degradation kinetics of RhB based on the experimental data in Figure 6a were calculated and shown in Figure 6b; all the photo-Fenton photocatalytic reactions followed the pseudo-first-order reaction kinetics, namely −ln(C/C_0_) = kt. In the above pseudo-first-order formula, t is the irradiation time, k is the apparent rate constants, and C_0_ and C represent the adsorption equilibrium concentration (at time = 0) and on-time concentration of RhB (at time = t), respectively. Then, a series of reaction rate constants (k) were obtained based on the pseudo-first-order reaction kinetic function, namely 0.030, 0.013, 0.037, 0.041, and 0.035 min^−1^, which correspond to g-C_3_N_4_, Cu-Cu_2_O, 5% Cu-Cu_2_O/g-C_3_N_4_, 30% Cu-Cu_2_O/g-C_3_N_4_, and 50% Cu-Cu_2_O/g-C_3_N_4_, respectively. It can be easily obtained that the 30% Cu-Cu_2_O/g-C_3_N_4_ catalyst exhibited the biggest k value (i.e., 0.041 min^−1^), indicating the best Photo-Fenton catalytic performance. Specifically, the reaction rate constants of 30% Cu-Cu_2_O/g-C_3_N_4_ were about 3 and 1.5 times higher than that of Cu-Cu_2_O and pure g-C_3_N_4_, respectively. The result further indicated that the Cu-Cu_2_O/g-C_3_N_4_ composite is a highly efficient photo-Fenton-like system as compared to g-C_3_N_4_ or Cu-Cu_2_O.

**Figure 7:**
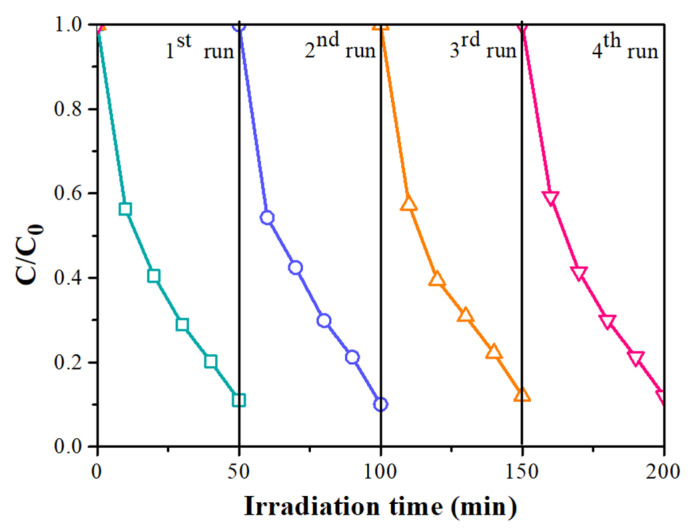
Cycling runs of 30% Cu-Cu_2_O/g-C_3_N_4_ catalyst for RhB degradation in the presence of H_2_O_2_ under visible light irradiation.

In addition, reusability is one of the most important indexes for the practical application of photocatalysts. Thus, cycling experiments using the 30% Cu-Cu_2_O/g-C_3_N_4_ catalyst were further conducted. As shown in Figure 7, the removal efficiency of RhB was only slightly decreased even after four runs, indicating the excellent stability of the 30% Cu-Cu_2_O/g-C_3_N_4_ photo-Fenton system.

**Figure 8:**
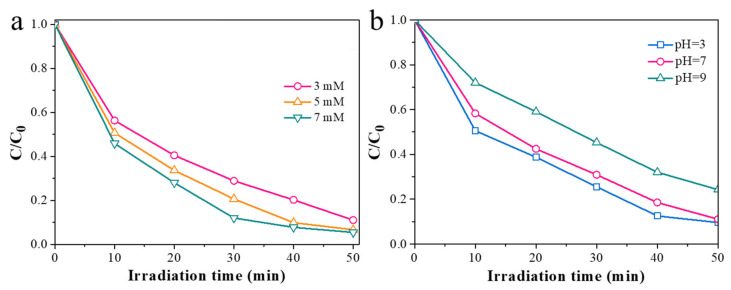
(**a**) Effect of initial concentration of H_2_O_2_ on degradation of RhB in suspensions. (**b**) Effect of initial pH on degradation of RhB with H_2_O_2_ (5 mM).

The effects of H_2_O_2_ dosage and pH value on the catalytic performance of 30% Cu-Cu_2_O/g-C_3_N_4_ were further studied. It can be observed in Figure 8a that only 10% of RhB was degraded after a reaction of 50 min in the absence of H_2_O_2_. When H_2_O_2_ was added, the degradation efficiency of RhB over the ternary samples was improved. Specifically, the degradation efficiency of RhB reached 90% when the content of H_2_O_2_ was at a low level (about 3 mM), and the degradation efficiency reached about 99% when the content of H_2_O_2_ increased to a medium level (about 5 mM). However, when the amount of H_2_O_2_ was more than 5 mM, the removal rate of RhB increased slightly since the H_2_O_2_ would act as a self-scavenger for ·OH. Figure 8b shows the effect of initial pH on RhB degradation in a 30% Cu-Cu_2_O/g-C_3_N_4_ photocatalytic system with 5 mM H_2_O_2_. As shown in Figure 8b, only a small drop-in degradation rate over the catalyst can be observed when the initial pH values ascended from 3 to 7. However, when the initial pH increased from 9, the degradation efficiency of RhB over the sample decreased dramatically. Generally speaking, in acidic conditions, a large number of H^+^ exists in the solution ions and it would promote the generation of ·OH, while in basic solution, OH^−^ ions could inhibit the formation of ·OH and further result in the decomposition of H_2_O_2_ to water and oxygen, thus resulting in the decrease in degradation efficiency [9,37].

### 2.3. Practical Applicability

**Figure 9:**
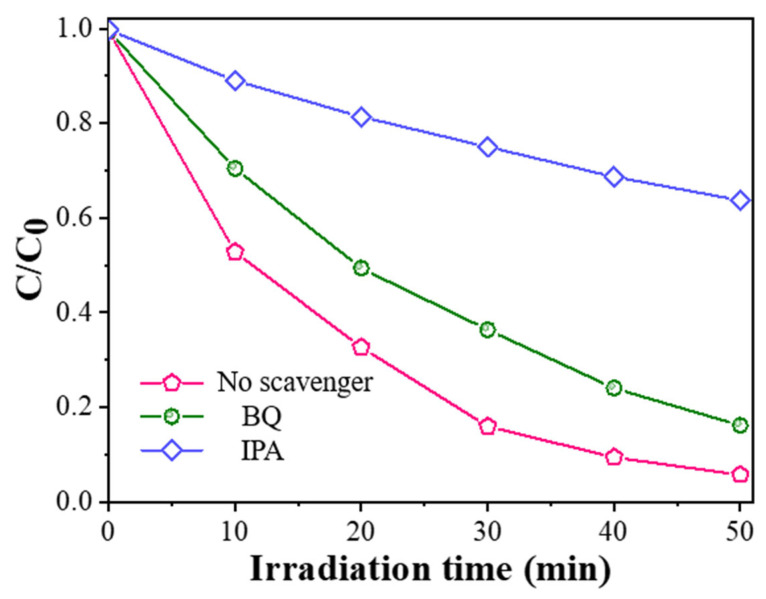
The effect of the different scavengers on RhB degradation over 30% Cu-Cu_2_O/g-C_3_N_4_ in the presence of H_2_O_2_ (7 mM) under visible light irradiation.

To further clarify the nature of the photo-Fenton photocatalytic process, the trapping experiments were used to explore the main active species. Different trapping reagents of isopropyl alcohol (IPA) and benzoquinone (BQ) were used to selectively react with the free radicals of ·OH and ·O_2_^−^, respectively. As shown in Figure 9, it is observed that the IPA affects the degradation much more compared to that of BQ, with a larger inhibition effect and a lower degradation rate of 36.3%, indicating that the main active species in the photo-Fenton reaction process are hydroxyl radicals.

**Scheme 1:**
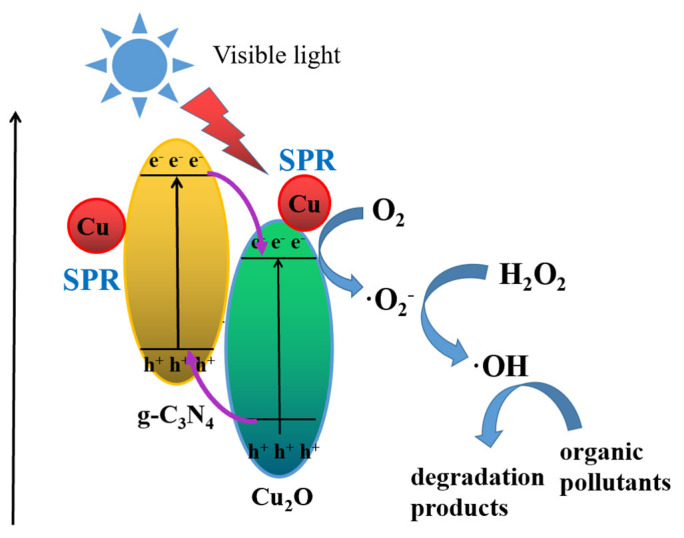
Schematic of the proposed photo-Fenton photocatalytic reaction mechanism for Cu-Cu_2_O/g-C_3_N_4_ composites (at pH = 7).

Based on the above experimental result, a possible reaction mechanism in the degradation of the RhB under visible light illustration for the Cu-Cu_2_O/g-C_3_N_4_-H_2_O_2_ system was proposed (Scheme 1). As it is known, g-C_3_N_4_ is an n-type semiconductor with a band gap of around 2.70 eV, and its conduction band (CB) and valence band (VB) potentials are −1.09 eV and +1.55 eV, respectively [38]. Cu_2_O is a p-type semiconductor with a band gap of 2.17 eV, and its CB and VB potentials are −0.28 eV and 1.92 eV, respectively [39]. Under visible light irradiation, both Cu_2_O and g-C_3_N_4_ in the ternary system can be excited. The photoinduced electrons will transfer from the CB of the g-C_3_N_4_ to that of Cu_2_O, while the photoinduced holes will accumulate to the VB of g-C_3_N_4_. As a result, photogenerated electron-hole pairs could be effectively separated, and the electrons accumulated on the CB of Cu_2_O, while the holes accumulated on the VB of the g-C_3_N_4_. Additionally, the photo-excited electrons generated from the plasmon-excited Cu metal (i.e., Cu^0^) could migrate to the conduction band of Cu_2_O (0.28 eV) due to the characteristic dipolar character of the SPR effect of Cu (Cu→Cu_2_O) [34]. It is accepted that the active sites (i.e., Cu^+^) in the Cu_2_O system will be oxidized and transformed to higher valance states (i.e., Cu^2+^) after a photo-Fenton-like reaction [8,37]. The accumulation of a large number of electrons on the CB of Cu_2_O can promote the reduction of Cu^2+^ to Cu^+^, therefore, an efficient catalytic cycle can be established under visible light irradiation.

According to the catalytic results, we can conclude that the addition of H_2_O_2_ can further enhance the catalytic performance of the Cu-Cu_2_O/g-C_3_N_4_ composite. It has been confirmed that the multiple valences of Cu serve as a photo-Fenton-like reagent and the g-C_3_N_4_ surface existence amino groups could accelerate the Fenton-like reaction in the absence of visible light irradiation [8,34]. Under visible light irradiation, the photo-induced electrons generated from the Cu-Cu_2_O/g-C_3_N_4_ system could activate H_2_O_2_ to form reactive species in the photo-Fenton-like reaction. Specifically, on the one hand, the photogenerated electrons could react with H_2_O_2_ and activate it to form ·OH species. On the other hand, the H_2_O_2_ could promote O_2_ to form an ·O_2_^−^ intermediate, and this intermediate was further converted to ·OH species. After the construction of a ternary system, the content of reactive ·OH formed in the reaction was increased; thus, the degradation efficiency of organic pollutants (i.e., RhB) was dramatically improved.

## 3. Materials and Methods

### 3.1. Materials

Cupric sulfate (CuSO_4_) and ammonium oxalate ((NH_4_)_2_C_2_O_4_) were purchased from Tianjin Damao’s chemical reagent. EDTA·2Na, urea, and H_2_O_2_ solution were obtained from Guangzhou Chemical Reagent Factory. Hydrazine hydrate, ammonia solution, and isopropyl alcohol were bought from Tianjin Zhi Yuan Reagent Co., Ltd. Benzoquinone was gained from Adames Reagent Co., Ltd. (Shanghai, China). All the chemicals used in this study are analytical grade and used as received without further purification.

### 3.2. Synthesis of g-C_3_N_4_ Nanosheets

Typically, 10 g of urea was added into a 50 mL alumina crucible, which contained 20 mL of distilled water, to prepare the pristine precursor solution. The crucible was put into an oven to re-crystallize at 70 °C until the water was completely dried out. Then, the sample was calcined at 550 °C for 2 h in a muffle furnace with a heating rate of 15 °C/min. Finally, the product (i.e., g-C_3_N_4_) was collected and then ground into powder for further use.

### 3.3. Preparation of the Cu-Cu_2_O/g-C_3_N_4_ Composition

In total, 0.2 g of g-C_3_N_4_ was dispersed into a beaker that contained 100 mL of distilled water, under constant stirring. Subsequently, a certain amount of CuSO_4_ and EDTA·2Na was added to the beaker. After 30 min stirring, 10 mL of hydrazine hydrate and ammonia solution (with a volume rate of 1:2) were added to the solution and kept stirring for another 30 min at room temperature. Then, the products were centrifuged and washed with distilled water and ethyl alcohol several times and then dried in a vacuum oven at 50 °C for 48 h. After the drying, the samples were calcined at 200 °C for 30 min in a muffle furnace. Consequently, Cu-Cu_2_O/g-C_3_N_4_ with the different mass ratios of Cu-Cu_2_O 5%, 30%, and 50% were prepared by changing the content of CuSO_4_. In addition, a bulk Cu-Cu_2_O sample was also synthesized without adding g-C_3_N_4_.

### 3.4. Characterizations Instrument

The physicochemical properties of as-obtained samples were characterized by a series of analytical methods. The crystal phase structure and composition of the prepared materials were analyzed by powder X-ray diffraction (XRD) measurements, which were performed on a Bruker D8 advanced X-ray diffractometer (Bruker AXS, Dresden, Germany) with a Cu kα radiation source. The surface morphologies were characterized by field emission transmission electron microscopy (TEM, JEM-2100HR, JEOL Ltd., Akishima-shi, Japan). The chemical compositions of the samples were determined by using X-ray photoelectron spectroscopy (XPS, ESCALAB 250, Thermo Fisher Scientific, Waltham, MA, USA). The UV–Vis diffuse reflection spectra (DRS) tests were performed on a UV–Vis spectrophotometer (UV-3010, Hitachi, Tokyo, Japan) using BaSO_4_ as a reference, and the measured data were converted from reflection to absorbance via the Kubelka–Munk method.

### 3.5. Photocatalytic Degradation

The photocatalytic degradation of RhB under different reaction conditions was carried out to evaluate the photocatalytic activity of the samples. Overall, 20 mg of as-prepared catalyst and 50 mL RhB solution were used in each experiment and were dispersed and the concentration of RhB solution was 10 mg/L. Before the photocatalytic tests, the suspension was placed in dark conditions for 30 min to reach the adsorption–desorption equilibrium. Thereafter, a certain quantity of 30% H_2_O_2_ was added to the above suspensions. In the tests, a 300 W Xe nom lamp (LTIC 300BE, Boyi Ltd., Guangzhou, China) with a 400 nm cut-off filter was used as a visible-light source. Under the continuous stirring at room temperature (25 °C), about 3 mL of solution was taken out at a specific moment of reaction time, and the catalysts were filtered by using a 0.45 μm nitrocellulose filter. The concentration of the filtered RhB solution was analyzed by a UV–Vis spectroscopy (UV-1800, Shimadzu, Shimadzu Coporation, Nishinokyo, Japan) at the wavelength of 553 nm.

### 3.6. Active Species Trapping Experiments

In this work, radical trapping experiments were carried out to confirm the main active species in the photocatalytic reaction. Specifically, in the degradation process of RhB, isopropyl alcohol (IPA, 1 mmol) and benzoquinone (BQ, 1 mmol) were separately injected into the solution to quench hydroxyl radicals (·OH) and superoxide radical anions (·O_2_^−^), respectively.

## 4. Conclusions

In summary, the different mass percentages of Cu-Cu_2_O combined with g-C_3_N_4_ catalysts have been successfully fabricated and used in photo-Fenton-like reactions. The results of catalytic experiments display that the Cu-Cu_2_O/g-C_3_N_4_ exhibits excellent performance in the wide pH range. In addition, a possible photo-Fenton photocatalytic reaction mechanism has been proposed based on the experimental results. The construction of a nanosheet-like Cu_2_O/g-C_3_N_4_ heterostructure with plasmonic metallic Cu would enhance the visible light absorption capacity and the separation efficiency of photo-generated charge carriers as well as the accumulation of electrons on the CB of Cu_2_O. The photo-excited electrons can promote the reduction of Cu^2+^ to Cu^+^, thus resulting in the restoration of the catalytic ability of the ternary catalysts in the photo-Fenton reactions. This work can provide a new solution to improve the activity of the g-C_3_N_4_-based catalyst in the photocatalytic reaction for organic pollutants degradation.

## Data Availability

Data are contained within the article.
